# Characteristics of traumatic major haemorrhage in a tertiary trauma center

**DOI:** 10.1186/s13049-024-01196-z

**Published:** 2024-03-25

**Authors:** Pieter van Wyk, Marcus Wannberg, Anna Gustafsson, Jane Yan, Agneta Wikman, Louis Riddez, Carl-Magnus Wahlgren

**Affiliations:** 1 Section of Acute and Trauma Surgery, Karolinska University Hospital, Stockholm, Sweden; 2 Department of Molecular Medicine and Surgery, Department of Vascular Surgery, Karolinska Institute, Karolinska University Hospital, SE-171 76 Stockholm, Sweden; 3 Department of Clinical Science, Intervention and Technology (CLINTEC), Karolinska Institute, Department of Clinical Immunology and Transfusion Medicine, Karolinska University Hospital, Stockholm, Sweden; 4 Division of Biostatistics, Institute of Environmental Medicine, Karolinska Institute, Stockholm, Sweden

**Keywords:** Trauma, Haemorrhage, Prehospital, Blood transfusion

## Abstract

**Background:**

Major traumatic haemorrhage is potentially preventable with rapid haemorrhage control and improved resuscitation techniques. Although advances in prehospital trauma management, haemorrhage is still associated with high mortality. The aim of this study was to use a recent pragmatic transfusion-based definition of major bleeding to characterize patients at risk of major bleeding and associated outcomes in this cohort after trauma.

**Methods:**

This was a retrospective cohort study including all trauma patients (*n* = 7020) admitted to a tertiary trauma center from January 2015 to June 2020. The major bleeding cohort (*n* = 145) was defined as transfusion of 4 units of any blood components (red blood cells, plasma, or platelets) within 2 h of injury. Univariate and multivariable logistic regression analyses were performed to identify risk factors for 24-hour and 30-day mortality post trauma admission.

**Results:**

In the major bleeding cohort (*n* = 145; 145/7020, 2.1% of the trauma population), there were 77% men (*n* = 112) and 23% women (*n* = 33), median age 39 years [IQR 26–53] and median Injury Severity Score (ISS) was 22 [IQR 13–34]. Blunt trauma dominated over penetrating trauma (58% vs. 42%) where high-energy fall was the most common blunt mechanism and knife injury was the most common penetrating mechanism. The major bleeding cohort was younger (OR 0.99; 95% CI 0.98 to 0.998, *P* = 0.012), less female gender (OR 0.66; 95% CI 0.45 to 0.98, *P* = 0.04), and had more penetrating trauma (OR 4.54; 95% CI 3.24 to 6.36, *P* = 0.001) than the rest of the trauma cohort. A prehospital (OR 2.39; 95% CI 1.34 to 4.28; *P* = 0.003) and emergency department (ED) (OR 6.91; 95% CI 4.49 to 10.66, *P* = 0.001) systolic blood pressure < 90 mmHg was associated with the major bleeding cohort as well as ED blood gas base excess < -3 (OR 7.72; 95% CI 5.37 to 11.11; *P* < 0.001) and INR > 1.2 (OR 3.09; 95% CI 2.16 to 4.43; *P* = 0.001). Emergency damage control laparotomy was performed more frequently in the major bleeding cohort (21.4% [*n* = 31] vs. 1.5% [*n* = 106]; OR 3.90; 95% CI 2.50 to 6.08; *P* < 0.001). There was no difference in transportation time from alarm to hospital arrival between the major bleeding cohort and the rest of the trauma cohort (47 [IQR 38;59] vs. 49 [IQR 40;62] minutes; *P* = 0.17). However, the major bleeding cohort had a shorter time from ED to first emergency procedure (71.5 [IQR 10.0;129.0] vs. 109.00 [IQR 54.0; 259.0] minutes, *P* < 0.001). In the major bleeding cohort, patients with penetrating trauma, compared to blunt trauma, had a shorter alarm to hospital arrival time (44.0 [IQR 35.5;54.0] vs. 50.0 [IQR 41.5;61.0], *P* = 0.013). The 24-hour mortality in the major bleeding cohort was 6.9% (10/145). All fatalities were due to blunt trauma; 40% (4/10) high energy fall, 20% (2/10) motor vehicle accident, 10% (1/10) motorcycle accident, 10% (1/10) traffic pedestrian, 10% (1/10) traffic other, and 10% (1/10) struck/hit by blunt object. In the logistic regression model, prehospital cardiac arrest (OR 83.4; 95% CI 3.37 to 2063; *P* = 0.007) and transportation time (OR 0.95, 95% CI 0.91 to 0.99, *P* = 0.02) were associated with 24-hour mortality.

**Results:**

Early identification of patients at high risk of major bleeding is challenging but essential for rapid definitive haemorrhage control. The major bleeding trauma cohort is a small part of the entire trauma population, and is characterized of being younger, male gender, higher ISS, and exposed to more penetrating trauma. Early identification of patients at high risk of major bleeding is challenging but essential for rapid definitive haemorrhage control.

**Supplementary Information:**

The online version contains supplementary material available at 10.1186/s13049-024-01196-z.

## Introduction

Trauma haemorrhage is considered the leading cause of potentially preventable deaths among injured patients [1,2]. Time is critical as haemorrhage is the most common cause of early death after arrival to a trauma centre [3, 4]. The median time to haemorrhagic death from hospital admission has been estimated to less than three hours, and approximately 85% of haemorrhagic deaths occur within six hours [5]. It is evident that early identification and control of haemorrhage both in the prehospital and early hospital phase is essential for the trauma management [6]. In addition to rapid transfer to definitive surgical care for bleeding control, prehospital resuscitation and the use of haemostatic agents may improve outcomes [7,8,9]. Although advances in prehospital trauma management with the use of haemostatic agents such as tranexamic acid and temporary bleeding control in extremity injuries with tourniquets, non-compressible truncal haemorrhage is still associated with high mortality [10]. Civilian and military studies suggest that early transfusion improves survival, but trial evidence remains inconclusive [11]. A post hoc analysis of two randomized clinical trials suggested that prehospital plasma is associated with a survival benefit when transport times are longer than 20 min [12]. However, a recent prospective multi-centre randomised controlled trial did not show that prehospital packed red blood cells and lyophilised plasma resuscitation was superior to 0.9% sodium chloride for trauma related haemorrhagic shock in the civilian population studied [13]. They concluded that future research should seek to identify if specific patient cohorts may benefit from prehospital blood transfusion and explore the effects of alternative transfusion strategies.

There is a need to better characterize patients at risk of acute major haemorrhage. The definition used in registry studies of major trauma bleeding has been variable and the traditional view of ten or more units of red blood cell (RBC) transfusion over 24 h has been questioned [14,15]. Accordingly, a clear and accepted definition of major traumatic bleeding for research purpose has been desirable [16]. Patients in traumatic haemorrhagic shock studies should also be monitored for secondary safety endpoints, including 24-hour and 30-day all-cause mortality [5].

The objective of this study was therefore to use a recent pragmatic transfusion-based definition of major bleeding to characterize patients at risk of major bleeding and associated outcomes in this cohort after trauma.

## Methods

### Study population and databases

This was a retrospective cohort study including all trauma patients admitted to Karolinska University Hospital Trauma Centre from January 1, 2015, to June 13, 2020. There were 7453 trauma patients registered during the study period. The study was approved by the Ethical Review Agency (Dnr 2020 − 01474). The STROBE (strengthening the reporting of observational studies in epidemiology) checklist for reporting cohort studies was used [17].

The population of Stockholm metropolitan area, approximately 6 500 km², and the nearby region is about 2.5 million. The prehospital service is provided by ground ambulances staffed by ambulance nurse and emergency medical technician as well as helicopter ambulance with nurse anaesthetist and physician-staffed rapid response vehicles providing advanced airway management.

Data were extracted from the trauma registry, SweTrau, the local hospital transfusion registry, and hospital medical records including anesthesia and operative charts. SweTrau, started in 2011 and is the only nationwide trauma database. The SweTrau database follows “*The Utstein Trauma Template for Uniform Reporting of Data Following Major Trauma; Data Dictionary*”, which represent a uniform set of variables considered most important for comparing trauma systems and outcomes in Europe [18]. Data access for this study was approved by the local registry steering committee.

The transfusion registry included data on exact issuing time and type of blood products ordered. The blood products are electronically reported to the registry when transfused. Blood products from trauma admission date and 24-hours thereafter were retrieved (*n* = 780). The registry has previously been validated as accurate for using as an alternative to manually retrieving the data from each of the patients’ medical records and anaesthesiology charts. Little inconsistency has been seen in issuing time and actual transfusion time; median 0.14 h (IQR 0.0-2.5) [19].

### Study variables

Major bleeding was defined as transfusion of 4 units of any (red blood cells, plasma, or platelets; whole blood was not provided during study period) blood components within 2 h of injury. This pragmatic transfusion-based definition of major bleeding was generated from a Delphi process and developed specifically for research purpose to guide registry data recording and to characterize patients at risk of major bleeding [16]. Both the Injury Severity Score (ISS) and the New Injury Severity Score (NISS) were used to classify trauma severity [20].

### Inclusion and exclusion criteria

At least one of the following inclusion criteria was needed for registration in Swetrau; trauma team activation, NISS > 15, or transferred from another hospital within 7 days. All patients who arrived in the ED with spontaneous circulation were included. Patients declared dead before hospital arrival, or those with no signs of life upon hospital arrival were not included in the registry. Patients < 18 years of age were excluded (*n* = 433).

**Fig. 1:**
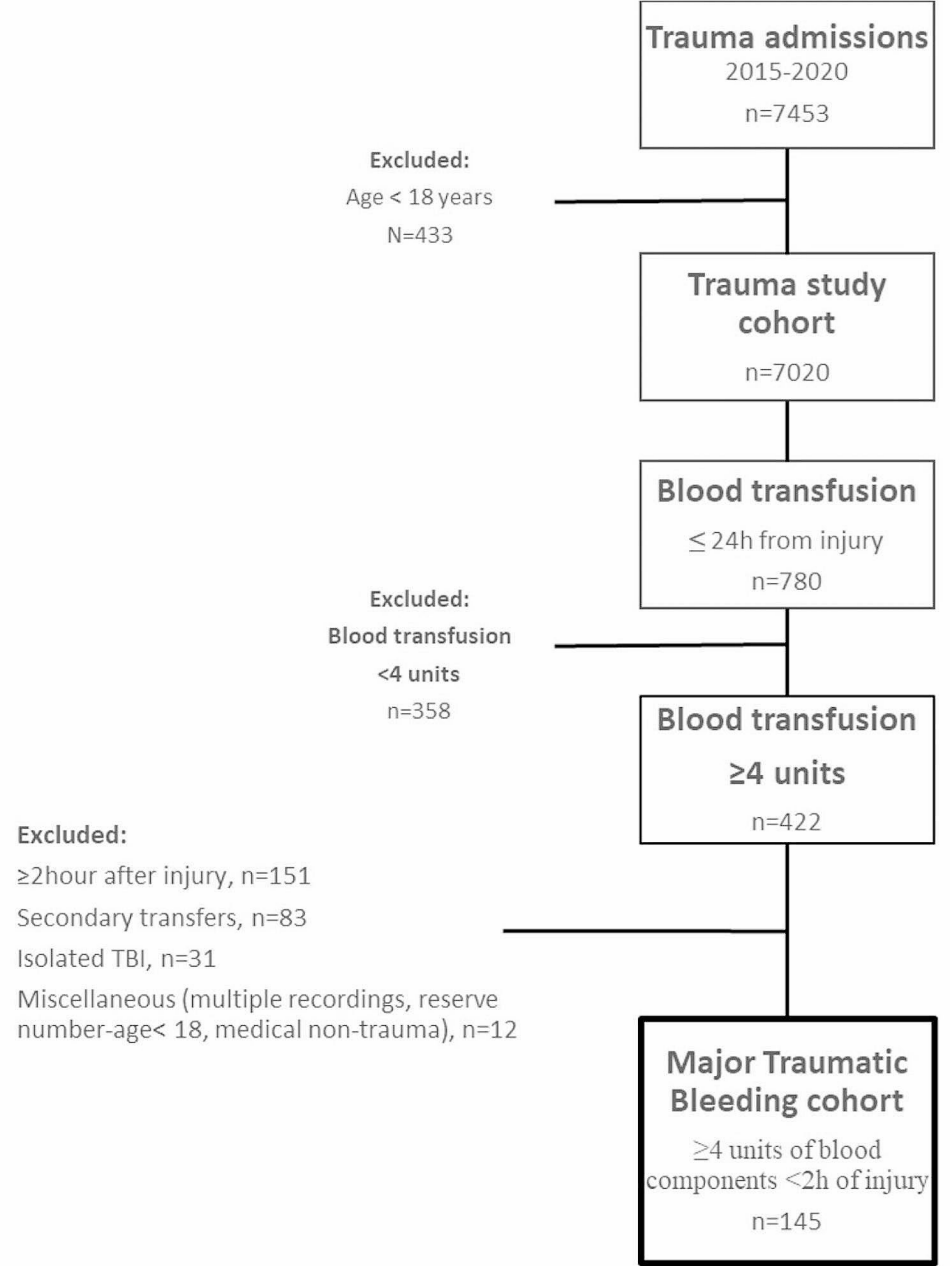
Flowchart patient inclusion

All adult patients admitted to the trauma center during the study period were included in the study (*n* = 7020) (Fig. 1, flowchart patient inclusion). There were 780 patients who received any blood product within the first 24 h after trauma, including 422 patients who receieved ≥ 4 units of any blood components. The final study cohort consisted of patients defined as major traumatic bleeding, receiving ≥ 4 units of any blood components within 2 h of injury (*n* = 145). Patients with secondary transfer and isolated traumatic brain injury were excluded from the final study cohort.

### Statistical analysis

Data were presented as median with interquartile range (IQR) for numeric variables and proportions for categorical variables. Descriptive statistics were performed for patient characteristics and outcomes. Logistic regression analyses were performed to compare the major bleeding cohort with the rest of the trauma cohort. Multivariable logistic regression analyses were performed to in the major bleeding cohort to identify associations to outcomes represented by 24-hour and 30-day mortality post trauma admission. The model fit was tested with the Hosmer-Lemeshow. Clinical relevant covariates, from available variables in the trauma registry, were included in the logistic analyses (age, gender, prehospital cardiac arrest, prehospital blood pressure < 90 mmHg, transportation times, and emergency surgical procedures). P-value < 0.05 was considered statistical significant. Analysis was performed with Stata MP 17.1.

## Results

### Patient demographics major bleeding cohort

In the major bleeding cohort (*n* = 145; 145/7020, 2.1% of the trauma population), there were 77% men (*n* = 112) and 23% women (*n* = 33), median age 39 years [IQR 26–53] (Table 1). The median ISS and NISS were 22 [IQR 13–34] and 27 [IQR 18–41], respectively. Blunt trauma dominated over penetrating trauma (58% vs. 42%) where high-energy fall (20%, 29/144) was the most common blunt mechanism and knife injury (27%, 39/144) was the most common penetrating mechanism (firearm 15%, 21/144). However, the proportions of penetrating (6.0%; blunt 1.4%) and firearm trauma (8.6%; knife 5.2%; high-energy fall 1.8%) were higher in the major bleeding cohort. The most common prehospital transportation was ground ambulance 74% (107/145) followed by helicopter 26% (37/145).

**Table 1:** Patient demographics and clinical data

		Major bleeding cohort	Trauma cohort	P-value
N		145	6875	
Age, median (IQR)		39.0 (26.0, 53.0)	44.0 (29.0, 61.0)	0.023
Gender	Male	112 (77.2%)	4760 (69.2%)	0.040
	Female	33 (22.8%)	2115 (30.8%)	0.040
Type of injury	Blunt	84 (57.9%)	5927/6874 (86.2%)	< 0.001
	Penetrating	61 (42.1%)	947/6874 (13.8%)	< 0.001
Prehospital cardiac arrest		6/144 (4.2%)	158/6059 (2.6%)	0.25
Prehospital GCS, median (IQR)		15 (12, 15)	15 (14, 15)	0.032
ED GSC, median (IQR)		15 (14, 15)	15 (15)	0.002
Prehospital SBP, median (IQR)		113 (95, 130)	130 (120, 150)	< 0.001
ED SBP, median (IQR)		120 (95, 140)	138 (123, 154)	< 0.001
Prehospital RR, median (IQR)		20 (18, 28)	18 (16, 22)	< 0.001
ED RR, median (IQR)		20 (17, 25)	18 (15, 20)	< 0.001
Prehospital Airway management		32/144 (22.2%)	390/6062 (6.4%)	< 0.001
ED intubation		61/144 (42.4%)	458/6873 (6.7%)	< 0.001
Prehospital transport	Ground ambulance	107 (73.8%)	5027/6272 (80.1%)	0.06
	Helicopter ambulance	37 (25.5%)	1035/6272 (16.5%)	0.004
	Private/public vehicle	0 (0.0%)	48/6272 (0.8%)	NA
	Walk-in	0 (0.0%)	102/6272 (1.6%)	NA
	Police	0 (0.0%)	25/6272 (0.4%)	NA
	Other	1 (0.7%)	35/6272(0.6%)	0.83
ISS, median (IQR)		22 (13, 34)	6 (1, 14)	< 0.001
NISS, median (IQR)		27 (18, 41)	9 (3, 20)	< 0.001
ED BE, median (IQR)		-4.20 (-7.20, -1.90)	0.60 (-1.80, 2.00)	< 0.001
ED INR, median (IQR)		1.10 (1.00, 1.20)	1.00 (1.00, 1.10)	< 0.001

ED-Emergency department, MVA-Motor vehicle accident, GCS-Glasgow Coma Scale, RR-Respiratory Rate, SBP-Systolic Blood Pressure, ISS-Injury Severity Score, NISS-New Injury Severity Score, BE-Arterial Base Excess, INR-International Normalized Ratio

### Clinical data major bleeding cohort

Prehospital and emergency department (ED) clinical data are displayed in Table 1. There was 9.0% (*n* = 13) and 19.3% (*n* = 28) with prehospital and ED systolic blood pressure < 90 mmHg, respectively. There were 4.1% (6/145) and 21.4% (31/145) of patients that underwent damage control thoracotomy or laparotomy, respectively, as first emergency operative procedure.

### Characteristics of major bleeding cohort vs. rest of the trauma cohort

The major bleeding cohort was younger (OR 0.99; 95% CI 0.98 to 0.998, *P* = 0.012), less female gender (OR 0.66; 95% CI 0.45 to 0.98, *P* = 0.04), and had more penetrating trauma (OR 4.54; 95% CI 3.24 to 6.36, *P* = 0.001) than the rest of the trauma cohort. A prehospital and emergency department systolic blood pressure < 90 mmHg was associated with the major bleeding cohort (OR 2.39; 95% CI 1.34 to 4.28; *P* = 0.003; OR 6.91; 95% CI 4.49 to 10.66, *P* = 0.001, respectively) as well as ED blood gas base excess < -3 (OR 7.72; 95% CI 5.37 to 11.11; *P* < 0.001) and INR > 1.2 (OR 3.09; 95% CI 2.16 to 4.43; *P* = 0.001). The prevalence of BE < -3 in the major bleeding cohort was 60% (76/127) (trauma cohort; 743/4594, 16%), and INR > 1.2 38% (50/132) (trauma cohort; 850/5163, 16%). Prehospital cardiac arrest was not specifically associated with the major bleeding cohort (OR 1.62; 95% CI 0.71 to 3.73; *P* = 0.25). Emergency damage control laparotomy was performed more frequently in the major bleeding cohort (21.4% [*n* = 31] vs. 1.5% [*n* = 106]; OR 3.90; 95% CI 2.50 to 6.08; *P* < 0.001).

### Transportation times in major bleeding cohort vs. the rest of the trauma cohort

There was no difference in transportation time from alarm to hospital arrival between the major bleeding cohort and the rest of the trauma cohort (47 [IQR 38;59] vs. 49 [IQR 40;62] minutes; *P* = 0.17). There was also no difference from trauma alarm to arrival at scene of injury (10.0 [IQR 7.0;14.0] vs. 10.0 [IQR 7.0; 15.0] minutes, *P* = 0.63). However, the major bleeding cohort had a shorter time from ED to first emergency procedure (71.5 [IQR 10.0;129.0] vs. 109.00 [IQR 54.0; 259.0] minutes, *P* < 0.001).

In the major bleeding cohort, patients with penetrating trauma, compared to blunt trauma, had a shorter alarm to hospital arrival time (44.0 [IQR 35.5;54.0] vs. 50.0 [IQR 41.5;61.0], *P* = 0.013).

### Major bleeding cohort and outcome

The median number of days on mechanical ventilation was three [IQR 1.0–12.0] and the median hospital length of stay was 12.5 days [IQR 5.5–26.5] which was significant longer compared to the rest of the trauma cohort (2.0 days [IQR 2.0–6.0; *P* < 0.001]. In-hospital death was higher in the major bleeding cohort (8.3%, 12/145, vs. 5.3%, 367/6875, *P* < 0.001).

The 24-hour mortality in the major bleeding cohort was 6.9% (10/145) (Table 2). All fatalities were due to blunt trauma; 40% (4/10) high energy fall, 20% (2/10) MVA, 10% (1/10) motorcycle accident, 10% (1/10) traffic pedestrian, 10% (1/10) traffic other, and 10% (1/10) struck/hit by blunt object. Prehospital cardiac arrest was more common in the mortality group (40%, 4/10, vs. 1.5%, 2/134, *P* < 0.001). There was no difference in transportation time (alarm to hospital; 50.5 (34–58) vs. 47 (38–60) minutes, *P* = 0.93). In the logistic regression model prehospital cardiac arrest (OR 83.4; 95% CI 3.37 to 2063; *P* = 0.007), but not age (OR 1.04; 95% CI 0.99 to 1.08; *P* = 0.10), female gender (OR 2.55; 95% CI 0.61 to 10.7; *P* = 0.20), or prehospital SBP < 90 mmHg (OR 0.99; 95% CI 0.24 to 4.09; *P* = 0.98), was associated with 24-hour mortality. There was an association between transportation time and 24-hour mortality (OR 0.95, 95% CI 0.91 to 0.99, *P* = 0.02).

The 30-day mortality in the major bleeding cohort was 9.0% (13/145) (Table 2). All fatalities were still due to blunt trauma (100% [13/13] vs. 54% [71/132]; *P* < 0.001) where high-energy fall (4/13) and MVA (4/13) dominated. In the logistic regression model, prehospital cardiac arrest was again associated with 30-day mortality (OR 36.3; 95% CI 2.01 to 655; *P* = 0.015).

**Table 2:** Outcome characteristics of major bleeding patients (*n* = 145) at 24-hour and 30-day

		24-hour outcome Dead (%)	Alive (%)			30-day outcome Dead (%)	Alive (%)
N (145)		10	135			13	132
Age, median (IQR)		38.5 (25–70)	39 (26–51)			58 (31–70)	38 (26–51
Gender	Male	6 (60)	106 (78.5)			8 (61.5)	104 (78.8)
	Female	4 (40)	29 (21.5)			5 (38.5)	28 (21.2)
Type of injury	Blunt	10 (100)	74 (54.8)			13 (100)	71 (53.8)
	Penetrating	0 (0)	61 (45.2)			0	61 (46.2)
Prehospital cardiac arrest		4 (40)	2/134 (1.5)			5 (38.5)	1/131 (0.8)
Prehospital SBP < 90 mmHg		2 (20)	11 (8.1)			3 (23.1)	10 (7.6)
Alarm-hospital, min (median, IQR)		50.5 (34–58)	47 (38–60)			56 (42–64)	47 (38–59)
ED emergency procedures	thoracotomy	4 (40)	2 (1.5)			4 (30.8)	2 (1.5)
	laparotomy	0	31 (23)			0	31 (23.5)

ED-Emergency department, MVA-Motor vehicle accident, SBP-Systolic Blood Pressure

## Discussion

Major traumatic haemorrhage is potentially preventable with rapid haemorrhage control and improved resuscitation techniques [21]. The time from injury to hospital, resuscitation, diagnosis, and definitive bleeding control should be as expeditiously as possible. Adult trauma patient with major bleeding requiring early blood transfusion, ≥ 4 units of any blood components within two hours from injury, constituted only of 2.1% of the trauma population admitted to hospital in this study.

The use of a transfusion-based definition of major bleeding within a short time frame from injury is in line with recent studies showing a median time to hemorrhagic death less than three hours [22,23,24]. The major bleeding definition from this Delphi process by Wong et al. also included all types of blood components to reflect a balanced approach to resuscitation [16]. This differs from historical bleeding definitions that are based on RBCs only [25].

It is imperative during the prehospital phase to identify those patients at high risk of severe haemorrhage [26]. The major bleeding cohort in this study was characterized of being younger, male gender, higher NISS, and exposed to more penetrating trauma compared to the rest of the trauma cohort. The physiology was also deteriorated with worse haemodynamics, and higher prevalence of acidosis and coagulopathy. There was no difference in transportation time from trauma alarm to arrival at scene of injury or from alarm to hospital arrival between the major bleeding cohort and the rest of the trauma cohort. This may indicate that prehospital teams need better strategies to identify patients requiring rapid transfer to definitive care. Additionally, when prehospital time is estimated to be long for logistical reasons, it may seem intuitive to provide prehospital blood products. During the study period, no prehospital blood products were given, but the access to prehospital blood is now available in the region. However, no clear recommendation according to recent European guidelines can be provided at this time in favour or against the use of prehospital blood products [27].

Patients with penetrating trauma had a shorter transportation time compared to blunt trauma in the major bleeding cohort. Early hospital blood transfusion indicated also a significant shorter time from ED to first emergency procedure where damage control laparotomy was performed more frequently. The importance of early haemorrhage control and decreased time to definitive surgical care has previously been showed [27]. In a study by Meizoso et al., delay to the operating room of more than ten minutes increased the risk of mortality by almost threefold in hypotensive patients with gunshot wounds [28].

The major bleeding cohort had a longer hospital course, and also higher in-hospital mortality (8.3%). More than ¾ of patients within the major bleeding mortality group, died within 24 h of injury where all fatalities were due to blunt trauma dominating by high energy falls and traffic accidents. Prehospital cardiac arrest was highly associated with mortality. For patients with major bleeding and blunt trauma, especially considering the longer transportation time for this cohort, there may be a potential benefit of prehospital blood transfusion resuscitation to preserve the patient’s circulating volume and maintain physiology [29]. There was no mortality in the major bleeding cohort after penetrating trauma which may be explained by the shorter transportation time and a clear indication for early in-hospital surgery.

There are limitations inherent to this study including limited variables or missing data due to lack of registration. There may be caveats using this definition of major bleeding. The exact time of injury may be challenging to determine, and we used in this study the time from the alarm. The time frame is short and patients with delays in transportation may not be captured. However, we believe that patients with severe critical bleeding with immediate need of surgical control in our urban context are mainly identified within this definition. The median transportation time, forty-seven minutes, seemed quite long for urban environment but shorter for penetrating trauma. This may be explained by prolonged patient extrication after high-energy falls and MVAs but also more obvious injuries and sources of bleeding may influence the prehospital personnel to prioritize short transfer time. There is also the possibility that traumatic haemorrhage may occur without reaching this transfusion criteria for major bleeding due to rapid surgical control, especially in penetrating trauma.

In conclusion, the major bleeding trauma cohort is a small part of the entire trauma population. This study has focused to characterize risk factors for early severe haemorrhage and associations to secondary endpoints. Early identification of patients at high risk of major bleeding is challenging but it is essential for this patient group to promptly initiate damage control resuscitation and surgery for definitive haemorrhage control.

### Electronic supplementary material

Below is the link to the electronic supplementary material.

Supplementary Material 1

## Data Availability

Please contact author for data requests.
