# Supplementary material for: Characteristics of traumatic major haemorrhage in a tertiary trauma center
Source: Scand J Trauma Resusc Emerg Med. 2024 Mar 25;32:24. doi: 10.1186/s13049-024-01196-z (PMC10962173; doi:10.1186/s13049-024-01196-z)
Supplement: Supplementary file 1 — Supplementary Material 1 [file 13049_2024_1196_MOESM1_ESM.docx]

**Dear Editor,**

We are happy for the potential acceptance decision. A revised manuscript has been submitted to the journal and comments from reviewers have been addressed. We are thankful for these comments and we believe the manuscript has improved.

Yours sincerely

Reviewer reports:


Reviewer #1:

Thank you very much review the revised manuscript «Characteristics of traumatic major haemorrhage in a tertiary trauma center» by van Wyk et al.

General:
Based upon the story (or manuscript) plot, the authors seek to identify major bleeding patients, as haemorrhage is associated with mortality. The hypothesis is that the identified patients could be treated in a different way, e.g. a more rapid transportation or a (what I perceive as your real primary study intention) prehospital blood transfusion.

The identification is based on a (non-standard) definition of major bleeding (4 unity of allogeneic blood products within 2 hours of injury). In this cohort patients were significantly younger, less female, had penetrating trauma, had more often a systolic blood pressure of blow 90 mmHg, had more often a metabolic acidosis and were more often coagulopathic. A cut off for "younger" is not given.

So the next step would be to perform an interventional study with this patient cohort ("young", male, coagulopathic, penetrating trauma, low blood pressure) to see if a different treatment would change patient outcome.

This story is somewhat constructed, and I find it difficult to distinguish between background, study plan and conclusion. I cannot identify the intention/plan of the authors in a consistent way. Why do they use a different or novel definition of massive transfusion? Why is this necessary? As I mentioned before, the identified cohort must undergo an interventional study to see the impact of an altered treatment plan. But is this the plan? The problem in trauma is: you cannot change the epidemics and the variables are (or may be) be dependent.

As mentioned in the first revision, maybe a comparison of multiple definitions of major haemorrhage (together with epidemiologic or laboratory variables) to better predict a worse outcome would be of a greater value.

**Thank you for these general comments. We believe there is a need to better characterize patients at risk of acute major haemorrhage. The definitions used in registry studies of major trauma bleeding have been variable and the traditional view of ten or more units of red blood cell (RBC) transfusion over 24 hours has been questioned. Recently, other transfusion endpoints have been developed and reported. However, these alternative definitions have not been widely applied internationally in the trauma literature. Accordingly, a clear and accepted definition of major traumatic bleeding for research purpose has been desirable.**

**With this in mind, our intention was to use this recent published transfusion-based definition of major bleeding to characterize patients at risk of major bleeding and associated outcomes. There was no study intention to compare this definition to older traditional bleeding definitions or to evaluate benefit of prehospital blood transfusion.**

**-The younger population in the major bleeding cohort refers to a significant younger age versus the overall trauma cohort (median age 39 vs. 44 y, P=0.023)**

The problem is that your work does not follow a straight plan. You have a lot of statistical analyses with "interesting" findings, but they do not allow your desired conclusion, and that is why the story plot is not concise. Your data does not allow any conclusion about prehospital blood transfusion. Association is not causation. I recommend: Present your data, conclude what can be concluded, do not speculate!

**Thank you. We believe the work follows the intended study plan but we appreciate the comment and we have tried in Results and Discussion to be more concise. Speculation is avoided and there is no conclusion about potential benefit of prehospital blood transfusion I selected patients (removed)**

Specific:
Statistical analysis: This section still seems insufficient to explain your statistical approach. E.g. why did you choose these covariates? Why did you include or exclude patients?

**The statistical section has been evaluated by our statistician. For the multivariable logistic regression analyses, clinical relevant covariates from the limited Utstein variables in the trauma registry were used. We have revised.**

**The inclusion/exclusion is described in figure 1. Patients for example with secondary transfers (different transportation time/blood transfusion at primary hospital) were excluded.**

Results:
What is the mortality group? Of course, resuscitation is associated with mortality. Why did you do this analysis? As mentioned before the statistical section is not sufficient to explain, why you did what kind of analysis. How is transportation time associated with 24h mortality? (longer time, higher mortality? Why is the transportation time longer?)

**Evaluation of secondary safety endpoints, including 24-hour and 30-day all-cause mortality, has been recommended when studying major traumatic bleeding (Fox/Holcomb Shock 2017). With the intention to better characterize the major bleeding cohort, and evaluate associations to 24h/30d-mortality, the logistic regression analysis was performed. The significant associations have been discussed in the Discussion section.**


Reviewer #2:

Thank you for a thorough and well-conducted revision of the manuscript. All my concerns have been considered and largely acted upon.

I have just one minor comment. The authors have revised the tables according to my suggestions, but to make the tables even more simple and clean, I suggest deleting the "no" group from both table 1 and 2 for prehospital cardiac arrest (table 1 and 2), prehospital airway management (table 1) and ED intubation (table 1). These "no" groups are just the opposite of the "yes" groups, and they don't add any additional information.

**Thank you.**

**Good comment. We have revised table 1 and 2 according to suggestions.**
